# Exploration of aminoacyl-tRNA synthetases from eukaryotic parasites for drug development

**DOI:** 10.1016/j.jbc.2022.102860

**Published:** 2022-12-31

**Authors:** Jasmita Gill, Amit Sharma

**Affiliations:** 1 ICMR-National Institute of Malaria Research, New Delhi, India; 2 Molecular Medicine Group, International Centre for Genetic Engineering and Biotechnology, New Delhi, India; 3 Academy of Scientific and Innovative Research (AcSIR), Ghaziabad, India

**Keywords:** *Plasmodium*, *Brugia*, *giardia*, *Toxoplasma gondii*, *leishmania*, *Cryptosporidium*, *trypanosoma*, aminoacyl-tRNA synthetases, drug discovery, aaRS, aminoacyl-tRNA synthetase, AlaRS, alanyl-tRNA synthetase, ArgRS, arginyl-tRNA synthetase, AsnRS, asparaginyl-tRNA synthetase, AspRS, aspartyl-tRNA synthetase, CysRS, cysteinyl-tRNA synthetase, GluRS, glutamyl-tRNA synthetase, HisRS, histidyl-tRNA synthetase, IleRS, isoleucyl-tRNA synthetase, LysRS, lysyl-tRNA synthetase, MetRS, methionyl-tRNA synthetase, PfArgRS, *Plasmodium falciparum* arginyl-tRNA synthetase, PheRS, phenylalanyl-tRNA synthetase, ProRS, prolyl-tRNA synthetase, ThrRS, threonyl-tRNA synthetase, TrpRS, tryptophanyl-tRNA synthetase, TyrRS, tyrosyl-tRNA synthetase, UBI, urea-based inhibitor

## Abstract

Parasitic diseases result in considerable human morbidity and mortality. The continuous emergence and spread of new drug-resistant parasite strains is an obstacle to controlling and eliminating many parasitic diseases. Aminoacyl-tRNA synthetases (aaRSs) are ubiquitous enzymes essential for protein synthesis. The design and development of diverse small molecule, drug-like inhibitors against parasite-encoded and expressed aaRSs have validated this enzyme family as druggable. In this work, we have compiled the progress to date towards establishing the druggability of aaRSs in terms of their biochemical characterization, validation as targets, inhibitor development, and structural interpretation from parasites responsible for malaria (*Plasmodium*), lymphatic filariasis (*Brugia,Wuchereria bancrofti*), giardiasis (*Giardia*), toxoplasmosis (*Toxoplasma gondii*), leishmaniasis (*Leishmania*), cryptosporidiosis (*Cryptosporidium*), and trypanosomiasis (*Trypanosoma*). This work thus provides a robust framework for the systematic dissection of aaRSs from these pathogens and will facilitate the cross-usage of potential inhibitors to jump-start anti-parasite drug development.

Multiple eukaryotic parasites are responsible for the prevalence and continuous spread of more than a billion infections worldwide, thus burdening public health initiatives and the economy (1, 2). Effective treatment and control of parasitic diseases needs the development of novel drugs as this process is further impeded by the periodic and inevitable development of drug resistance in parasites, and insecticide resistance in vectors. Such barriers to effective treatment could permit the resurgence of parasitic diseases, so there is an urgent need for novel anti-parasite drug scaffolds.

Eukaryotic parasites can cause diverse diseases of varying severity in hosts, including both animals and humans. The parasites *Plasmodium*, *Toxoplasma gondii*, and *Cryptosporidium* of the phylum Apicomplexa are responsible for causing malaria, toxoplasmosis and cryptosporidiosis respectively (1, 2, 3). *Plasmodium* species are responsible for the most acute forms of infection in humans after the proliferation and killing of red blood cells, with estimated 241 million malaria cases in 85 endemic countries (1, 2, 3) (Fig. 1). *Plasmodium vivax* is more geographically widespread than *Plasmodium falciparum* and both are responsible for causing severe infections but only the former for relapses. *T. gondii*, an intracellular parasite, infects animals; however, they are also pathogenic in immunocompromised humans and can cause infections *via* food-borne illnesses (4). This parasite is estimated to persist chronically in 25 to 30% of the global population (4). After the human hosts ingest cysts, sporozoites are released. These sporozoites infect epithelial cells of the intestine, where the sporozoites develop into tachyzoites which multiply and infect more cells (Fig. 1). These stages together account for some symptoms of the disease (4) (Fig. 1), and limited drugs are available for the treatment of toxoplasmosis. *Cryptosporidium* affects bovine calves by infecting epithelial cells of the intestine, causing gastrointestinal disease leading to severe and chronic diarrhea. It can further cause direct or indirect human exposure and have debilitating effects, especially in immunocompromised individuals (5). *Cryptosporidium hominis* and *Cryptosporidium parvum* are known to cause intestinal infections in humans (Fig. 1).

**Figure 1:**
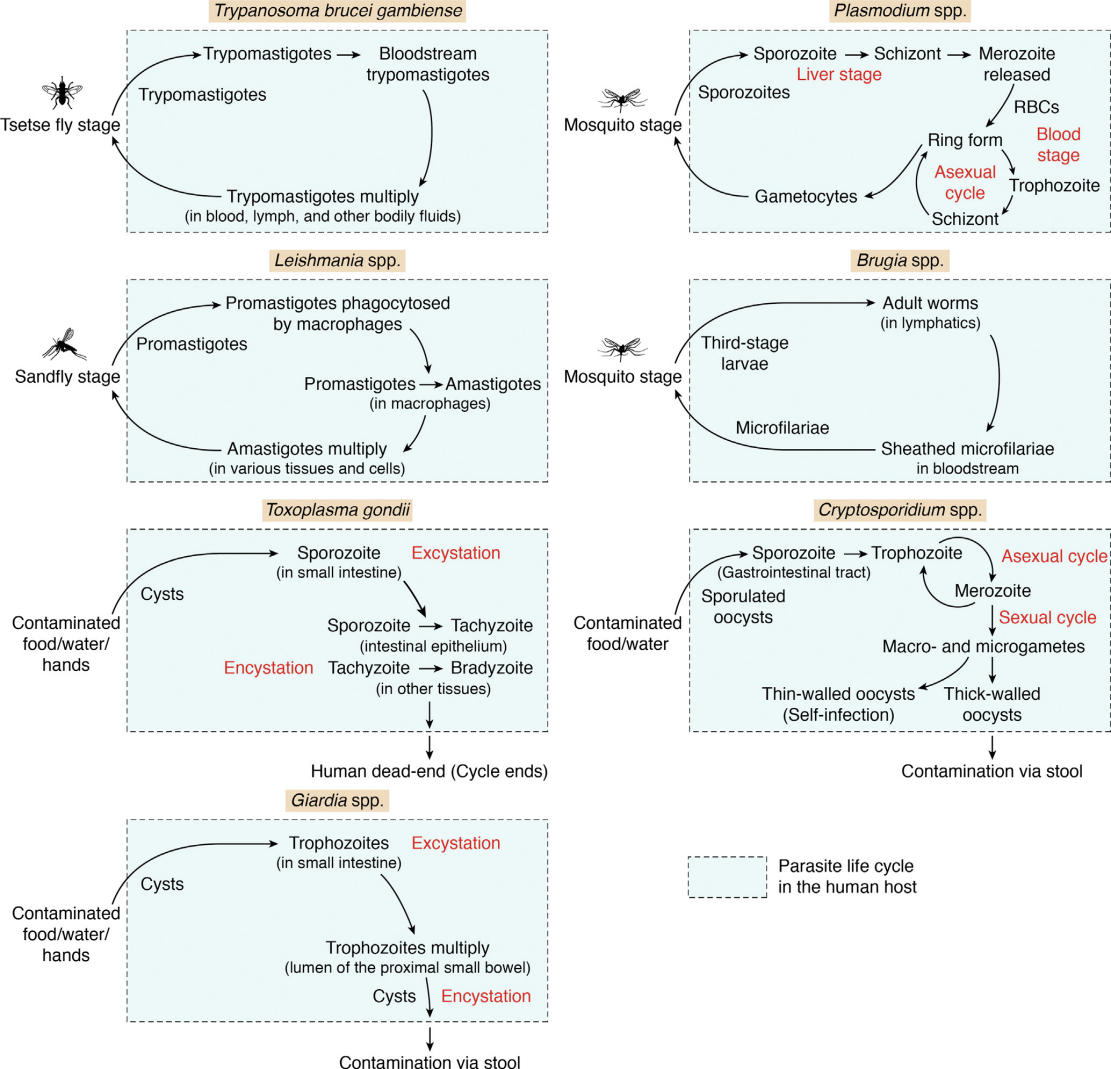
**Life cycle of eukaryotic parasites in the human host**. *Trypanosoma brucei gambiense*: The infected tsetse fly injects metacyclic trypomastigotes into the host’s bloodstream where they transform into bloodstream trypomastigotes. The trypomastigotes multiply through binary fission in blood, lymph, and other body fluids and are ingested by the tsetse flies that bite the infected human host. *Plasmodium spp.*: The infected female *Anopheles* mosquito, while taking a blood meal, injects sporozoites into the human host. The sporozoites infect the liver cells where they mature into schizonts. Upon the rupture of these schizonts, merozoites are released into the bloodstream. The merozoites infect the red blood cells and mature into rings, trophozoites and schizonts, which rupture and release more merozoites into the bloodstream. Some merozoites mature into male and female gametocytes which the mosquito ingests when it takes blood meal from an infected host. *Leishmania spp.*: The female sandfly injects promastigotes into the human bloodstream while taking a blood meal. The promastigotes are phagocytosed by immune cells like macrophages. Once inside the macrophage, the promastigote matures into amastigote. The amastigote multiplies inside these cells through division and, upon rupturing of the cells, infects other cells. The amastigotes are ingested by sandflies when they take blood meal from an infected human. *Brugia spp*.: The third-stage *Brugia* larvae enter the human host *via* bites of *Mansonia* and *Aedes* mosquitoes. The larvae develop into adult worms in the human lymphatic system. The adult worms produce microfilariae which enter the bloodstream and are ingested by mosquitoes when they take a blood meal. *Toxoplasma gondii*: Humans are an intermediate host for *Toxoplasma gondii*. They are infected by the ingestion of *T. gondii* cysts, mostly through contaminated food. Upon ingestion, the excystation occurs, and sporozoites are released. These sporozoites infect the intestinal epithelial cells, where the sporozoites develop into tachyzoites. The tachyzoites undergo asexual reproduction and go on to infect more cells. Some of these tachyzoites invade tissue systems, forming bradyzoites through encystation. The bradyzoites can remain undetected in the human host for a long time, becoming active only when the host’s immune status is compromised. *Cryptosporidium spp*.: The sporulated oocyst of *Cryptosporidium* can enter the human host through the ingestion of fecally contaminated water or food. The oocyst releases sporozoites in the gastrointestinal (GI) tract. The parasites infect the epithelial cells in the GI tract and undergo asexual (producing schizonts and merozoites) and sexual (producing and macro- and micro-gametes) life cycles. Following the fertilization of gametes, the zygote produces two kinds of oocysts: thick-walled and thin-walled. The thin-walled oocysts continue infecting cells within the host, whereas the thick-walled oocysts are transmitted into the environment through faeces. *Giardia spp.*: The *Giardia* cysts enter human hosts through the oral ingestion of contaminated food or water. The cysts shed their external hard cover in the small intestine and release trophozoites which remain in the lumen. The trophozoites replicate through longitudinal binary fission. Some of them form cysts which are infectious and are passed in the stool.

Similarly, trypanosomatid parasites *Trypanosoma* and *Leishmania* of phylum Euglenozoa are two important parasites that cause human diseases (6, 7). *T. brucei* causes African trypanosomiasis (sleeping sickness), where they proliferate inside the bloodstream and the human lymphatic system and subsequently affect the central nervous system often leading to fatality (Fig. 1). *Trypanosoma cruzi* causes the chronic and fatal Chagas disease which affects 6 to 7 million people worldwide. Leishmaniasis is one of the neglected tropical diseases (7). The two *Leishmania* parasites *Leishmania major* and *Leishmania donovani* are responsible for human infections (Fig. 1) of varying severity, causing cutaneous leishmaniasis and visceral leishmaniasis (kala-azar) with an estimated 700,000 to 1 million new cases annually. Cutaneous leishmaniasis causes skin sores, while visceral leishmaniasis, the serious form of the disease, causes injury to internal organs (7). There is a lack of effective drugs for these Trypanosomatid parasites, compounded by the threat of the emergence of resistance to available drugs. Other eukaryotic parasites with anaerobic metabolism, like *Giardia* (causes giardiasis), *Trichomonas* (trichomoniasis), and *Entamoeba* (amebiasis), are also a public health problem (8). Although nitroimidazole drugs can be used, resistance remains a significant issue. The helminth parasites *Brugia malayi* and *Wuchereria bancrofti* cause lymphatic filariasis (elephantiasis) in humans, which is triggered by the immune system’s reaction to adult worms and can lead to permanent disability (9) (Fig. 1). In this work only *Brugia* parasite will be discussed. The treatment of such diverse parasitic diseases urgently requires the identification of robust drug targets and the continued development and design of novel drugs in order to tackle drug resistance. One such family of essential enzymes, the aminoacyl-tRNA synthetases (aaRSs), which tend to be conserved within different parasites, hold promise as a target for anti-parasite drug development.

## aaRSs as antiparasitic drug targets

**Figure 2:**
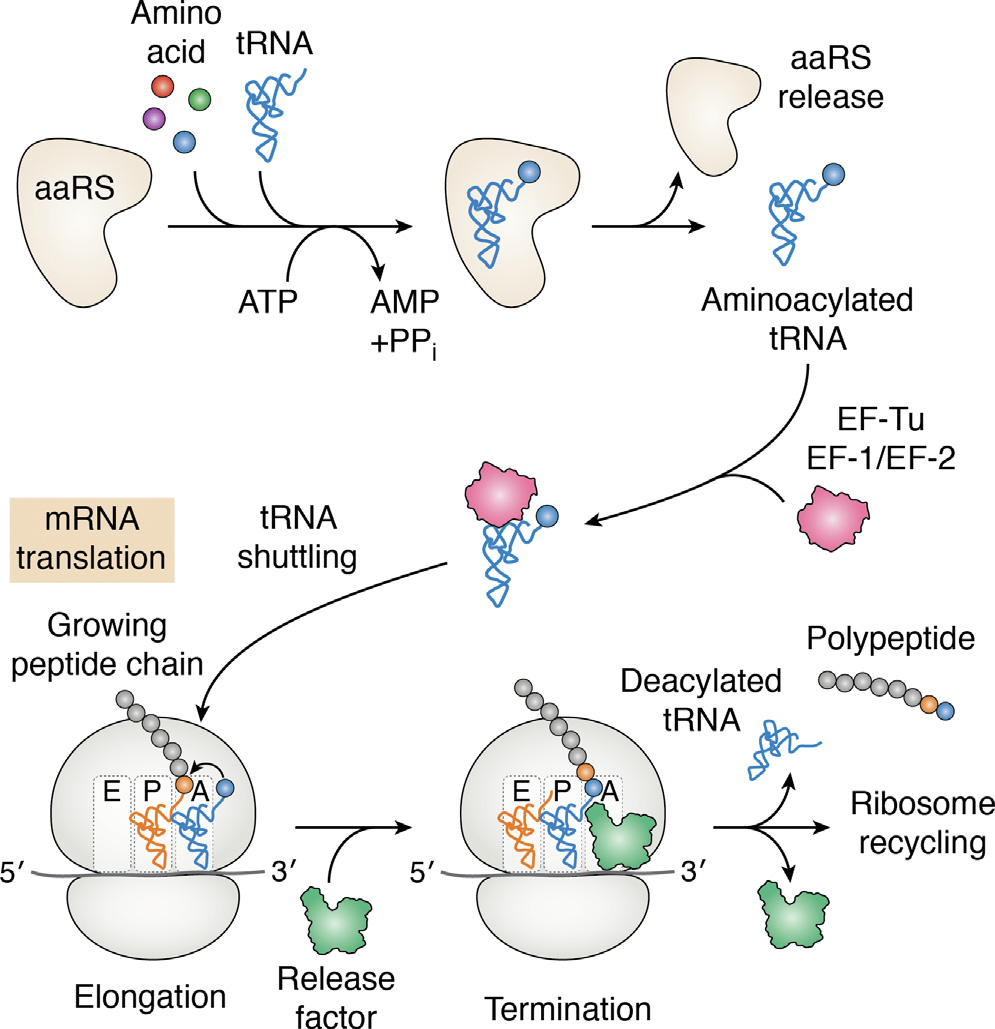
**The critical role of aminoacyl-tRNA synthetases in protein synthesis.** Aminoacyl-tRNA synthetases (aaRSs) charge tRNAs with the corresponding amino acids. These aminoacylated tRNAs bind to elongation factors for transport to the ribosome. At the A-site of the ribosome, aminoacylated tRNAs bind and recognize the presented codon on the mRNA by base pairing. Then, the nascent protein chain is transfered from a tRNA located at the ribosome’s P-site, thereby elongating the chain by one amino acid. A stop codon at the A-site triggers the accommodation of a release factor instead of a tRNA, which leads to release of the polypeptide chain (*i.e.* termination).

**Figure 3:**
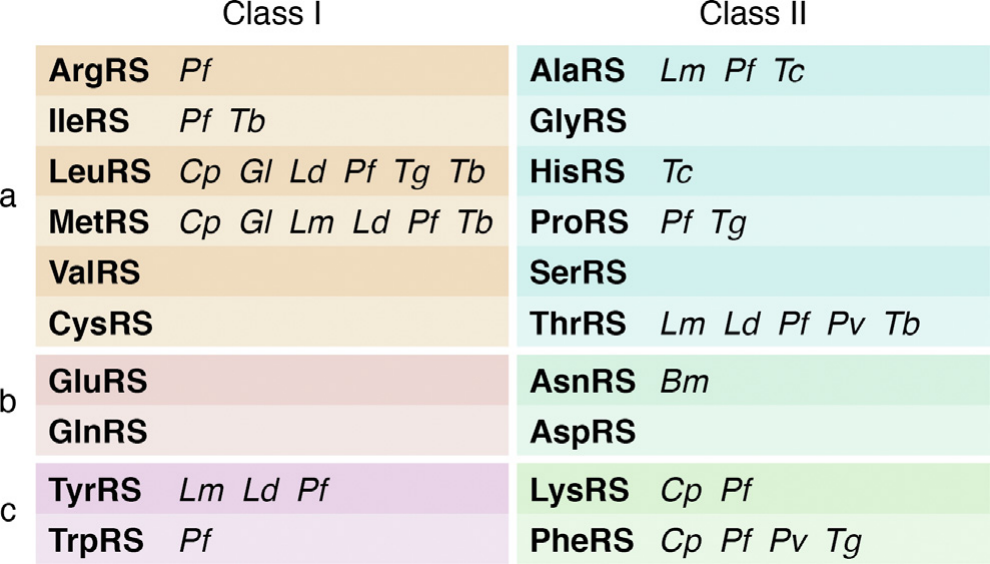
**Classification of aminoacyl-tRNA synthetases into class I and II and subclasses a, b and c.** The parasites for which inhibitors have been developed against specific aaRSs are listed; *Cp*: *Cryptosporidium parvum*, *Tb*: *Trypanosoma brucei*, *Tc*: *Trypanosoma cruzi*, *Pf*: *Plasmodium falciparum*, *Pv*: *Plasmodium vivax*, *Tg*: *Toxoplasma gondii*, *Gl*: *Giardia lamblia*, *Bm*: *Brugia malayi*, *Lm*: *Leishmania major*, *Ld*: *Leishmania donovani*. aaRS, aminoacyl-tRNA synthetase.

**Figure 4:**
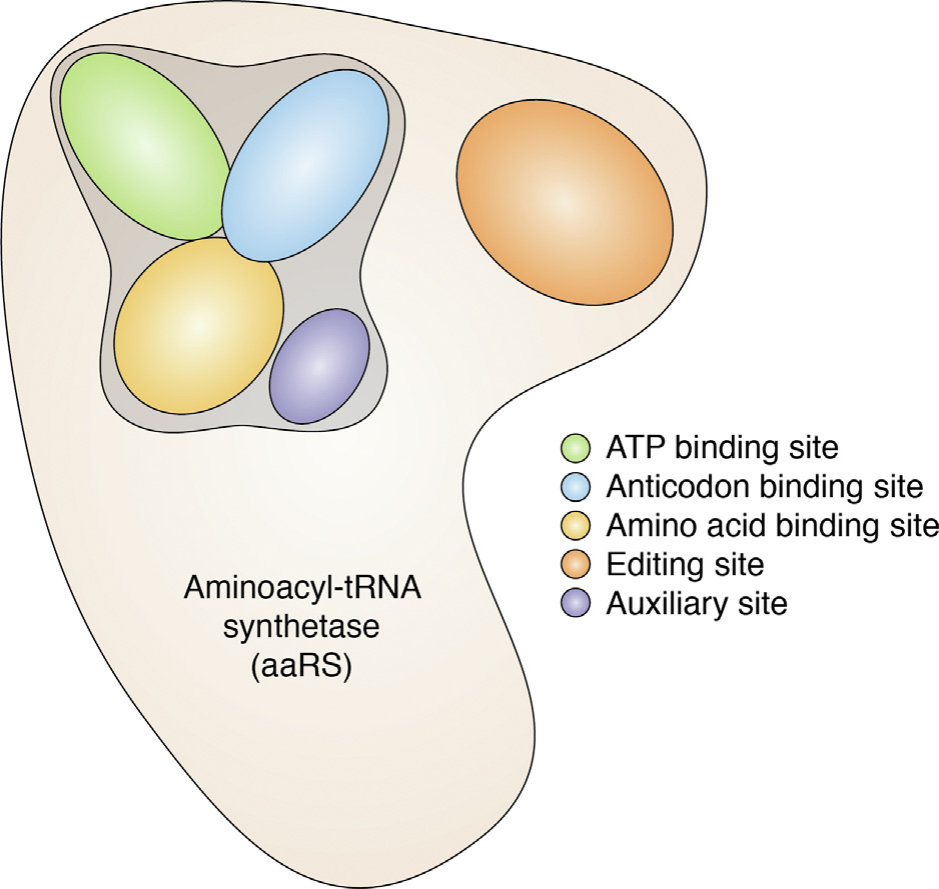
**The potential druggable sites on aminoacyl-tRNA synthetases.** The sites for likely interaction between aaRS (belonging to either class I or II) and a compound/inhibitor/drug which can inhibit the enzyme activity are highlighted; 1. ATP-binding site, 2. anticodon-binding site, 3. amino-acid–binding site, 4. editing site, and 5. auxiliary site.

The aaRSs family of enzymes (also known as aminoacyl-tRNA ligases) are ubiquitous since they catalyze the linking of cognate amino acid that corresponds to the tRNA anticodon triplet (2, 10, 11, 12, 13) (Fig. 2). The enzymatic reaction comprises of two steps; first, aaRS utilizes an ATP molecule to activate the cognate amino acid to generate an active aminoacyl-adenylate intermediate (amino acid-AMP) releasing pyrophosphate (PPi). Second, the cognate tRNA binds to the enzyme, which transfers the amino acid to the 3′ end of tRNA, releasing AMP (1, 10, 11, 12, 13). The resulting aminoacylated tRNA, an essential substrate for protein translation, is then transported by elongation factors to the ribosome to carry out protein synthesis (Fig. 2). Aminoacyl-tRNA synthetases also contain editing domains that ensure high fidelity of tRNA charging (12, 13). The aaRSs reduce errors by hydrolyzing misactivated amino acids (pretransfer to the tRNA) (Fig. 2) and misacylated tRNAs utilizing separate posttransfer-editing domains (11). Aminoacyl-tRNA synthetases are thus essential enzymes for protein synthesis (i) for providing aminoacylated-tRNA with the cognate amino acid and (ii) for ensuring the accuracy of protein translation (Fig. 2). The aaRSs are also important for several other cellular processes beyond their catalytic roles, including regulation of transcription, biosynthesis of signal molecules, and mitochondrial RNA cleavage (10, 11, 12, 13). The aminoacyl-tRNA synthetases are categorized into two classes, I and II, on the basis of their structure, where class I aaRSs are mostly monomeric and contain the Rossman fold catalytic domain (2, 12, 13) (Fig. 3). On the other hand, class II has a characteristic antiparallel beta-sheet fold surrounded by alpha-helices. Aminoacyl tRNA synthetases are prominently conserved in their catalytic domain due to their specific function; however, their sequence, structure, and function are seen to be relatively diverse across species. Structural and experimental data show that eukaryotic parasite aaRSs enzymes are excellent drug targets with multiple druggable sites; an ATP-binding pocket, the adjoining amino acid–binding pocket, and a tRNA recognition site (2, 10, 11, 12, 13) (Fig. 4). The editing domains that are present on some aaRSs are additional targets for drugs. Some parasite aaRSs are localized to the cytosol (also simply referred to as the cytoplasm) and another subcellular organelle, apicoplast, a vestigial nonphotosynthetic plastid. The apicoplast is essential for parasite survival as it plays a crucial role in lipid metabolism in malaria parasites. The parasites are dependent on the apicoplast and on the mitochondria, and some aaRSs are dual localized in the cytosol and the apicoplast (2, 3, 4, 5, 6, 7, 8, 9, 10, 11, 12, 13). Thus, aaRSs in multiple organelles are potential drug targets in parasites.

Aminoacyl-tRNA synthetases are well-established drug targets for antibacterial and antifungal activities (14, 15, 16). Several inhibitors have been developed, of which an antibiotic, the isoleucyl-tRNA synthetase (IleRS) inhibitor mupirocin, and the LeuRS inhibitor tavaborole, which is an antifungal, are approved for clinical treatment of methicillin-resistant *Staphylococcus aureus* and fungal-infective onychomycosis (14, 15, 16). As drug targets, aaRSs have promising potential because the parasite, like all life forms, is reliant on protein translation. Moreover, due to the specific requirement of active and fast proliferation, parasites are sensitive towards disruption in the critical machinery of protein translation.

## Inhibitors against parasite aminoacyl-tRNA synthetases

In this work we summarize advancements in exploring parasite aminoacyl-tRNA synthetases as drug targets by consolidating experimental data on biochemical characterization, validation, inhibitor development, and three-dimensional structural dissections for aminoacyl-tRNA synthetases (in alphabetical order starting from alanyl-tRNA synthetase (AlaRS)) from seven eukaryotic pathogens *Brugia* spp., *Cryptosporidium* spp., *Giardia* spp., *Leishmania* spp., *Plasmodium* spp., *T. gondii*, and *Trypanosoma* spp. This work will facilitate research integration and provide new directions for antipathogen drug discovery.

### Alanyl-tRNA synthetase

**Figure 5:**
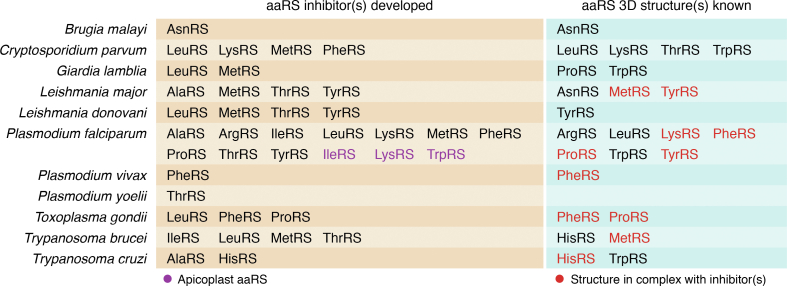
**Inhibitors developed and three-dimensional structures of aminoacyl-tRNA synthetases from eukaryotic parasites (January 2006 till June 2022)**. The cytoplasmic aaRSs are shown in *black font* where AlaRS corresponds to Alanyl-tRNA synthetase. The apicoplast aaRSs are shown in *purple font*. *Red font color* denotes where three-dimensional structure is known in complex with an inhibitor. aaRS, aminoacyl-tRNA synthetase; AlaRS, Alanyl-tRNA synthetase.

A single nuclear gene in the parasite *Plasmodium* encodes for AlaRS, giving rise to two proteins with different localizations, that is, the cytosol and the apicoplast (17, 18). *Plasmodium* AlaRS also contains a second active site with editing activity since glycine and serine are the most common mischarging events due to their similar size (19). AlaRS presents an opportunity to target aminoacylation and the editing activities occurring in two distinct parasite compartments. Several potential *P. falciparum* AlaRS inhibitors were screened *in silico* using homology models, revealing one compound A5, (4-{2-nitro-1-propenyl}-1,2-benzenediol), that was validated to inhibit parasite growth at micromolar levels while producing sparse cytotoxicity (Table 1) (Figure 3, Figure 4, Figure 5) (18). In another study, a pre-validated MNP library (marine natural product; a specific group of bacterial extract prefractions with demonstrated activity against *Leishmania*) was used and four potential *L. major* AlaRS inhibitors decreased the overall tRNA-AlaRS aminoacylation activity (20, 21). The three promising mixes (1881C, 2059D, and 2096D) affected aminoacylation with inhibition ranging from 80% to 99% (Table 1). Interestingly, cross-reactivity is also seen with *T. cruzi* AlaRS, which indicates a broad-spectrum potential and no effect on the human homolog (20). AlaRS is yet to be explored in four of the seven pathogens *Brugia*, *Cryptosporidium*, *Giardia* and *Toxoplasma,* discussed in this review (20). The first three-dimensional structure of parasite AlaRS remains to be determined; however, similar to *Plasmodium*, homology-modeled structures from bacteria and fungi could be explored for *in silico* docking.

**Table 1:** Inhibitors developed for aminoacyl-tRNA synthetases against eukaryotic parasites up till June 2022

aaRSs	Inhibitor(s)	Parasite	Binding mechanism	Reference
AlaRS	A3; A5	*Plasmodium falciparum*	Active site a	Khan et al., 2011 (18)
	Natural marine product library (1881C, 2059D, and 2096D)	*Leishmania major* *Trypanosoma cruzi*	Active site a	Kelly et al., 2020 (20)
ArgRS	hemin	*Plasmodium falciparum*	Not known	Jain et al., 2016 (22)
AsnRS	Variolin B	*Brugia malayi*	Active site a	Sukuru et al., 2006 (27)
	Natural product extracts (L-aspartate-B-hydroxamate	*Brugia malayi*	Pretransfer editing site a	Danel et al., 2011 (28)
	TAM B (from *Streptomyces* sp. 17944 extracts)	*Brugia malayi*	Pretransfer editing site a	Yu et al., 2011 (29)
	WS9326D (from *Streptomyces* sp. 9078 extracts)	*Brugia malayi*	Pretransfer editing site a	Yu et al., 2012 (30)
	Adipostatins A-D (from *Streptomyces* sp. 4875 extracts)	*Brugia malayi*	Pretransfer editing site a	Rateb et al., 2015 (31)
HisRS	15 fragments	*Trypanosoma cruzi*	Auxiliary site	Koh et al., 2015 (33)
IleRS apicoplast	Mupirocin	*Plasmodium falciparum*	Active site a	Istvan et al., 2011 (35)
IleRS	Thiaisoleucine	*Plasmodium falciparum*	Active site a	Istvan et al., 2011 (35)
	NSC70422 (Ile-AMP analog)	*Trypanosoma brucei*	Active site a	Cestari and Stuart 2013 (36)
LeuRS	Benzoxaborole derivatives	*Trypanosoma brucei*	Post-transfer editing site	Ding et al., 2011 (37)
	2-Pyrrolinone derivatives	*Trypanosoma brucei*	Active site (predicted; 3D model of active site)	Zhao et al., 2012 (40)
	N-(4-sulfamoylphenyl)thioureas derivatives	*Trypanosoma brucei*	Active site (predicted; 3D model of active site)	Zhang et al., 2013 (41)
	3,5-dicaffeoylquinic acid and derivatives	*Giardia lamblia*	Active site a	Zhang et al., 2012 (42)
	AN2690	*Leishmania donovani*	Active site a	Minhas et al., 2018 (39), Tandon et al., 2020 (38)
	Benzoxaborole derivatives (AN6426, AN8432)	*Plasmodium falciparum*	Editing active site	Sonoiki et al., 2016 (43)
	Benzoxaborole derivative (AN6426)	*Cryptosporidium parvum Toxoplasma gondii*	Editing active site	Palencia et al., 2016 (44)
	Series of α-phenoxy-*N* sulfonylphenyl acetamides (Compound 28g)	*Trypanosoma brucei*	Active site (predicted; 3D model of active site)	Xin et al., 2020 (45)
	Amides (Compound 74 and 91)	*Trypanosoma brucei*	Active site (predicted; 3D model of active site)	Li et al., 2021 (46)
LysRS	Cladosporin	*Plasmodium falciparum*	Active site	Khan et al., 2014 (50)
	Compound 5	*Plasmodium falciparum Cryptosporidium parvum*	Active site	Baragana et al., 2019 (52)
	ASP3026 (anaplastic lymphoma kinase inhibitor)	*Plasmodium falciparum*	Active site	Zhou et al., 2020 (56)
	Cladosporin derivatives, CL-2	*Plasmodium falciparum*	Active site	Babbar et al., 2021 (54)
	Cladosporin derivatives, Cla-B, Cla-C	*Plasmodium falciparum*	Active site	Babbar et al., 2021 (55)
LysRS2 apicoplast	M-26, M-37	*Plasmodium falciparum*	Active site a	Hoen et al., 2013 (51)
MetRS	Aminoquinoline derivatives (Compound 1)	*Trypanosoma brucei*	Active site (predicted model)	Shibata et al., 2011 (57)
	Urea-based inhibitor series (Compound 2 and 26)	*Trypanosoma brucei*	Active site (predicted model)	Shibata et al., 2012 (59)
	Series of urea-based inhibitors (UBIs)	*Trypanosoma brucei*	Active site	Koh et al., 2014 (60)
	MSMLR library of small molecules: 12 compounds	*Trypanosoma brucei*	Active site a	Pedro-Rosa et al., 2015 (62)
	Fluro-imidazopyridine (Compound-1717)	*Giardia intestinalis* *Giardia lamblia*	Not known	Ranade et al., 2015 (67) Michaels et al., 2020 (68)
	REP3123 and REP8839, C1, C2, C3	*Plasmodium falciparum*	Active site (predicted; *in silico*)	Hussain et al., 2015 (65)
	Ursolic acid	*antileishmanial* *antitrypanosomal*	Active site (predicted; *in silico*)	Labib et al., 2016 (63)
	Imidazopyridine-containing compounds (2093, 2114, 2259)	*Cryptosporidium parvum* *Cryptosporidium hominis*	Active site a	Buckner et al., 2019 (66)
	Compound 1 and 26	*Trypanosoma brucei*	Active site	Zhang et al., 2020 (61)
	DDD806905	*Leishmania major*	Allosteric ligand-binding site	Torrie et al., 2020 (69)
PheRS	Bicyclic azetidines (BRD7929, BRD8494)	*Cryptosporidium parvum*	Active site and an auxiliary site	Funkhouser-Jones et al., 2020 (71)
	Bicyclic azetidine (BRD7929)	*Cryptosporidium parvum*	Active site a	Vinayak et al., 2020 (70)
	Bicyclic azetidine (BRD1389)	*Plasmodium vivax*	Active site	Sharma et al., 2021 (75)
	Bicyclic azetidine (BRD7929)	*Toxoplasma gondii*	Active site	Radke et al., 2022 (72)
		*Plasmodium falciparum*	Active site	Sharma et al., 2022 (74)
ProRS	Halofuginone	*Plasmodium falciparum*	Active site	Herman et al., 2015 (76) and Jain et al., 2014 (80)
	Halofuginol (derivative of halofuginone)	*Plasmodium falciparum* *Plasmodium berghei*	Active site a	Herman et al., 2015 (76)
	Halofuginone	*Toxoplasma gondii*	Active site	Jain et al., 2015 (78)
	Febrifugine and halofuginone derivatives	*Plasmodium falciparum*	Active site	Jain et al., 2014 (80) Jain et al., 2017 (81)
	1-(pyridin-4-yl) pyrrolidin-2-one derivatives	*Plasmodium falciparum*		Okaniwa et al., 2021 (83)
	Double drugging: halofuginone and ATP analog L95	*Toxoplasma gondii*	Active site	Manickam et al., 2022 (82)
ThrRS	Borrelidin	*Plasmodium falciparum Plasmodium yoelii*	Active site a	Otoguro et al., 2003 (86)
	Borrelidin analogs	*Plasmodium falciparum*	Active site a	Suguwara et al., 2013 (87)
	T1-T11	*Plasmodium falciparum*	Active site a	Khan et al., 2011 (18)
	Borrelidin	*Trypanosoma brucei*	Active site a	Kalidas et al., 2014 (88)
	Borrelidin	*Leishmania donovani*	Active site a	Chadha et al., 2018 (89)
	Natural marine product library (1438C, 1758C, 2059D, and 2096B)	*Leishmania major*	Active site a	Kelly et al., 2020 (20)
TrpRS apicoplast	Indolmycin	*Plasmodium falciparum*	Active site a	Pasaje et al., 2016 (95)
TyrRS	Fisetin	*Leishmania major* *Leishmania donovani*	Active site	Larson et al., 2011 (96) Anand et al., 2016 (97)
	ML901	*Plasmodium falciparum*	Active site	Xie et al., 2022 (100)

a **Predicted binding mechanism:** The inhibitor has been experimentally validated to inhibit aminoacylation/pretransfer editing activity of the aaRS enzyme and thus is predicted to bind at the active/pretransfer-editing site. However, structural interpretation or validation is not available.

### Arginyl-tRNA synthetase

*P. falciparum* cytoplasmic arginyl-tRNA synthetase (*Pf*ArgRS) is a class I monomeric enzyme. IC_50,_ a half-maximal inhibitory concentration, measures the potency of a compound in inhibiting enzyme activity. Hemin, an iron-containing porphyrin, binds *Pf*ArgRS and inhibits its aminoacylation activity with IC_50_ of ∼2 μM (Table 1) (22). Hemin induced a dimeric form of *Pf*ArgRS, making it inactive and thus incapable of recognizing the cognate tRNA^Arg^. Increased levels of hemin, particularly in chloroquine-treated malaria parasites, led to decreased levels of tRNA^Arg^. At the same time, the human ArgRS can recognize the tRNA^Arg^ even in the presence of hemin. However, the binding site of hemin on the three-dimensional structure of *Pf*ArgRS is unknown. ArgRS has been explored only in *P. falciparum* and is among the least studied aaRSs. The structure of *Pf*ArgRS can be utilized for homology modeling and docking in the other six pathogens discussed, and aminoacylation activity inhibition can be tested using established approaches (22).

### Asparaginyl-tRNA synthetase

The cytoplasmic AsnRS was first characterized as a druggable target in the nematode *B. malayi* (23). *Bm*AsnRS catalyzes the production of diadenosine triphosphate, and binding studies involving *Bm*AsnRS show a possible role of AsnRS in modulating immune cell function (24, 25). The solution structure of *B. malayi* AsnRS revealed a lysine-rich region in its N-terminus, which interacts with tRNA (26).

*In silico* docking followed by experimental testing of docked compounds against *B. malayi* AsnRS revealed 45 compounds with mid-micromolar IC_50_s (27). A marine natural product called Variolin B inhibited ∼50% of *Brugia* AsnRS activity at 50 μM concentration (Table 1) (27). *Bm*AsnRS can recognize and edit misacylation before the transfer to tRNA, and thus a “pre-transfer assay” identifies compounds and allows for screening inhibitors. Natural product extracts (L-aspartate-B-hydroxamate, an asparagine analog) were identified using this approach (Table 1) (28). TAM B (isolated from *Streptomyces* sp 17944 extracts), belonging to the tirandamycins class of compounds was shown to kill adult *B. malayi* parasites (29) (Fig. 1). Another study from the same group revealed a compound WS936D from *Streptomyces* sp 9078 extracts (WS9326A derivatives) that inhibits *B. malayi* AsnRS aminoacylation, thereby killing adult *B. malayi* parasites at low nanomolar concentrations (30). These compounds did show selectivity, as no notable cytotoxicity was observed in human hepatic cells (30). Another study discovered four novel alkylresorcinols called adipostatins A, B, C, and D (from active strain *Streptomyces* sp. 4875), which all inhibit *B. malayi* AsnRS with apparent IC_50_s estimated at 15 μm for adipostatins A, B, and C and 30 μM for adipostatin D (31). They also kill adult parasites *in vitro* without any notable general cytotoxicity in host cells (Table 1) (31). AsnRS remains to be explored in six of the seven pathogens discussed, and only apo structure is known from *Brugia*. The efficient high-throughput screening platform with recombinant *B. malayi* could be reoriented for other pathogens (31).

### Histidyl-tRNA synthetase

The first structures of eukaryotic histidyl-tRNA synthetase (HisRS) were determined from *T. cruzi* and *T. brucei* (32). While the *T. cruzi* structure was apo, *T. brucei* HisRS is a complex with L-His and histidyladenylate, wherein the binding interactions are vastly distinct from bacterial or human homologs. Upon L-His binding, a rearrangement occurs in the active site, which was not significant during the formation of the first product histidyladenylate after L-His reacts with ATP (32). Fifteen newly identified fragments (from a library of 680) are structurally bound in a new “fragment-binding pocket” in *T. cruzi* HisRS, which is essentially a narrow groove proximal to the bound L-His (Table 1) (33, 34). It is suggested that the fragments likely compete for binding with ATP or the product HAMP or possibly both, causing inhibition of HisRS. This pocket can potentially achieve the desired “selectivity” since this pocket is absent in human HisRS. However, low affinities of these fragments warranted very high concentrations, which is not desirable; thus, enzyme inhibition has to be considered with caution (33). Nevertheless, these fragments can be utilized as a starting point for developing inhibitors of trypanosomatid HisRS and for the other six pathogens discussed, in which HisRS has not been explored as a drug target yet. Available structures of *T. cruzi* and *T. brucei* HisRS can be explored to address the significance of the “fragment-binding site”.

### Isoleucyl-tRNA synthetase

Mupirocin, an established drug against bacterial IleRS, inhibits the *P. falciparum* growth in the blood stage in a nanomolar range (35) (Fig. 1). This study analyzed *P. falciparum* parasites resistant to mupirocin that have mutations in their apicoplast IleRS, validating it as a drug target (35). Also, the cytoplasmic *Plasmodium* IleRS was inhibited by isoleucine analog thiaisoleucine. Both mupirocin and thiaisoleucine showed the elimination of cultured parasites *in vivo* (Table 1) (35). Twenty small molecules were identified from compounds available from National Cancer Institute that were similar to the intermediate Ile-AMP. These could kill *T. brucei* forms in the bloodstream (Table 1) (36) (Fig. 1). Compound NSC70422 notably showed good selectivity against mammalian cells and cured *T. brucei*–infected mice with low cell toxicity as it acted as a competitive inhibitor of the *Tb*IleRS (36). IleRS is yet to be explored in five of seven pathogens. The three-dimensional structure of parasite IleRS is unavailable, but structures of bacterial and fungal IleRS can be used for *in silico* docking.

### Leucyl-tRNA synthetase

A modeled structure of *T. brucei* Connective Polypeptide 1 (editing) domain based upon *Candida albicans* LeuRS was utilized to develop several benzoxaborole compounds, since AN2690 (5-fluro-1.3-dihydro-1-hydroxy-2,1-benzoxaborole) has been used as an antifungal successfully against *C. albicans* (37). These compounds also inhibited *T. brucei* LeuRS aminoacylation activity by targeting the LeuRS-editing site. Further, *ex vivo* growth was inhibited at low micromolar IC_50_s with negligible host toxicity (Table 1) (37). Similarly, the Connective Polypeptide 1 domain of *L. donovani* LeuRS was critical for editing the mischarged tRNA and aminoacylation activity (38). AN2690 also had a low-to-moderate affinity to *Ld*LeuRS (K_d_ = 30 μM) as it inhibits parasite growth *in vitro* and *in vivo* in BALB/c mice while exhibiting negligible toxicity in host cells (39). Zhao *et al*. revealed a novel set of compounds with a 2-pyrrolinone scaffold by *in silico* screening (SPECS chemical library) of modeled *Tb*LeuRS active site (Table 1) (40). Another novel class of *Tb*LeuRS inhibitors (N-(4-sulfamoylphenyl)thioureas), similarly targeting a 3D *in silico* model of the synthetic active site, were identified by screening and then modifying the small, targeted library of potential aaRSs inhibitors (41). They mimic the intermediate aminoacyl-AMP; however, most compounds had poor permeability and poor inhibitions except compound 59, which had IC_50_ = 1.1 μM. In a separate study, 3,5-dicaffeoylquinic acid and its derivatives, including 3,5-dicaffeoylquinic acid propyl ester (viz., compounds 2, 3, and 4), were good at killing the *Giardia lamblia* parasites with IC₅₀ values of 1.79, 5.51, and 2.56 μM, respectively (Table 1) (42) and the derivatives notably exhibited reduced toxicity and enhanced activity. These were an aqueous ethanol extract of dicaffeoylquinic acids containing *Artemisia argyi* (42). Similarly, in a separate study, two 3-aminomethyl compounds (termed AN6426 and AN8432) were potent against multidrug-resistant *P. falciparum* W2 strain with 50% inhibitory concentration (IC_50_s: 310 nM and 490 nM) (Table 1) (43). The treatment was effective against *Plasmodium berghei* infection after oral administration once a day for 4 days in a murine model.

AN6426 also inhibits growth in human cells for *C. parvum* and *T. gondii*. Similar inhibition activity is seen against *Cryptosporidium* and *Toxoplasma* parasites as it targets the LeuRS-editing site (Table 1) (44). In *T. gondii*, it prevents the proliferation of *Toxoplasma* parasites in human fibroblasts at mid-micromolar concentrations. Also, their activity intensifies in the presence of amino acid norvaline which can be mischarged to tRNA^Leu^ and is a substrate for post-transfer editing by LeuRS. AN6426 and tRNA^Leu^ form a covalent adduct in the enzyme’s editing site, either blocking aminoacylation if it interacts with the tRNA^Leu^ acceptor end or blocking post-transfer editing if it interacts, for example, with ATP (44). Recent studies identified a series of α-phenoxy-*N* sulfonylphenyl acetamides as inhibitors of *T. brucei* LeuRS using a 3D *in silico* model of the active site. Compound 28 g was the most potent, with an IC_50_ of 0.70 μM, and potency higher by 250-fold than the starting hit, that is compound 1 (Table 1) (45). In a subsequent study, utilizing the initial hit compound thiourea ZCL539, a follow-up series of amides were designed and synthetized and proven effectual against *T. brucei* LeuRS. Compounds 74 and 91 were the most potent compounds with IC_50_ of 0.24 and 0.25 μM (about 700-fold higher potent than the starting hit) (Table 1) (46). LeuRS and its inhibitors that target the editing active site are well-studied in all seven pathogens discussed except *Brugia*, and apo structure is available from *P. falciparum* for *in silico* approaches.

### Lysyl-tRNA synthetase

The natural product cladosporin, a fungal secondary metabolite, is active against blood and liver stage growth of *P. falciparum* at a nanomolar range. Cladosporin targets the cytosolic lysyl-tRNA synthetase (LysRS), as parasites that overexpress LysRS are resistant to cladosporin (47). The apo structure of LysRS from *Entamoeba histolytica* in a complex with small ligands shows that conformational changes occur upon lysine binding in the catalytic domain, similar to the earlier reports on bacterial LysRS structures (48). Three-dimensional structures of *Pf*LysRS in apo form and complex with substrates show cladosporin interacting with the ATP-binding site as it mimics the natural substrate adenosine (49, 50). *In silico* docking revealed two potent compounds, M-26 and M-37, showing delayed death inhibition by inhibiting aminoacylation activity by recombinant *P. falciparum* apicoplast LysRS (Table 1) (51). Baragana *et al.* reported selective inhibitors of the apicomplexan LysRS, of which compound 5, when given at a low oral dosage (1.5 mg/kg once daily for 4 days), reduced parasitemia by over 90% in a malaria mouse model as it also inhibits *C. parvum* LysRS and growth of *C. parvum* parasites *in vitro* (Table 1) (52). In addition, compound 5 reduced the parasite burden by almost two times when given orally for 7 days in two separate mouse models of cryptosporidiosis (52). This study also reported the 3D structure of *C. parvum* LysRS in complex with cladosporin and L-Lys. LysRS-1 (KRS-1) from *L. donovani* is an essential gene (53).

Cladosporin, due to its poor bioavailability and high metabolic instability, is unable to progress toward being a drug candidate. It became appropriate to explore analogs of cladosporin against *Pf*LysRS with slight stereochemical or functional modifications. Four sets of analogs designed by making modifications in the scaffold of cladosporin were assessed in enzyme and parasite assays (Table 1) (54). The most potent compound, CL-2, performed better than cladosporin, and additional H-bonds were noted along with increased aqueous solubility. CL-2 binds to the adenosine pocket itself. The IC_50_ for CL-2 was 0.1 μM in the ATP hydrolysis assay, and the EC_50_ values for CL-2 were 0.08 and 4.0 μM (54). EC_50_ measures the concentration of the compound to obtain a 50% killing in a cell-based assay. The same group later reported another set of derivatives of cladosporin, Cla-B and Cla-C, where the tetrahydropyran frame was replaced with a piperidine ring having functional implications (55). Complex structures with Cla-B and Cla-C reveal similar binding orientations as *Pf*LysRS with cladosporin bound (Table 1) (55). However, the orientation of the piperidine ring varies from that of the tetrahydropyran ring of the cladosporin. Screening of 1215 bioactive compounds led to the discovery of ASP3026, an anaplastic lymphoma kinase inhibitor, as a *Pf*LysRS inhibitor with nanomolar potency and > 80-fold more effective than the human LysRS (Table 1) (56). ASP3026 occupies the same site as cladosporin with few structural adjustments. ASP3026 is already used in clinical trials against B-cell lymphoma and solid tumors (56). LysRS is among the well-studied aaRSs from *P. falciparum* and *C. parvum*, thus providing a robust platform to explore LysRS in the other pathogens.

### Methionyl-tRNA synthetase

*T. brucei* methionyl-tRNA synthetase (MetRS) is an essential enzyme as its gene knockout showed growth defects (57). Further, several effective compounds showed more than 95% inhibition of aminoacylation activity at 50 nM concentration (57). Compound 1 was most effective in *T. brucei* mouse model with delivery at 25 mg/kg/day for 3 days showing high parasite suppression and delayed death and low mammalian cell toxicity (Table 1) (Fig. 1). Subsequently, *L. major* MetRS complexed with products methionyladenylate and pyrophosphate showed significant rearrangements in the overall structure of *Lm*MetRS and/or tRNA (as compared to bacterial MetRS) that are vital to enable tRNA^Met^ to access the methionyladenylate intermediate at the active site (58).

In the first major study to develop improved inhibitors of *T. brucei* MetRS, urea-based scaffolds held promise, firstly due to increased bioavailability and secondly, their likely permeability through the blood-brain barrier (Table 1) (59, 60). Urea-based compounds inhibited parasite growth with low EC_50_ values (0.15 μM) and low toxicity to host cells. Compounds 2 and 26 showed superior membrane permeation in the *in vitro* MDR1-MDCKII model (which predicts and classifies compounds with blood barrier permeability) and improved oral pharmacokinetic properties in mice. Compound 26 also showed good suppressive activity against *T. brucei rhodesiense* in the mouse model, and compound 2 was seen to have entered the central nervous system in mice (59). Subsequently, several urea-based inhibitors (UBIs) were designed against *Tb*MetRS having IC_50_ of ∼19 nM (Table 1) (60). UBIs bind to *Tb*MetRS through conformational selection and very optimal binding in two pockets—the L-Met pocket and another conserved auxiliary pocket which is likely to be involved in tRNA binding. The UBIs do not compete with ATP for binding but rather interact with it *via* an h-bond. Thus, the omnipresent ATP-binding mode of MetRSs can be employed to design inhibitors for other disease-causing pathogens (60). A beneficial fluorination site for inhibitors targeting *T. brucei* MetRS was identified after the structural evaluation of *Tb*MetRS complexes (Table 1) (61). One series of compounds has a 1,3-dihydro-imidazol-2-one containing linker, while a second series includes a rigid fused aromatic ring. These distinct series inhibit parasite growth with high potency and EC_50_< 19 nM with low toxicity in mammalian cells. Selectivity was achieved in the range of 20- to 200-fold (61). 5-fluoroimidazo[4,5-b]pyridine, when incorporated into compounds, imparts bioavailability and improved efficacy (61). In another study, a large number of inhibitors of *Tb*MetRS was identified (1270) from MLSMR library by BioFocus DPI containing small molecules in molecular weight range 350 to 410 g/mol and containing both natural and synthetic products (Table 1) (62). Fifty two of fifty four compounds chosen for low-throughput screening were active in *T. brucei* aminoacylation activity assay. Twelve of the fifty four hit compounds inhibit the growth of *T. brucei* in culture, most likely *via* inhibition of *Tb*MetRS (62).

Ursolic acid, a natural derivative taken from a reliable source of fresh leaves of *Ochrosia elliptica Labill*., of family Apocynaceae, displays potent antitrypanosomal and antileishmanial activities (Table 1) (63). The IC_50_ values were encouraging, between 1.53 and 8.79 μg/ml and almost the same as that of pentamidine, an existing treatment for leishmaniasis though it has many side effects. Ursolic acid exhibited considerable affinity to MetRS with free binding energies from −42.54 to −63.93 kcal/mol (63). Further, two new compounds containing the tetracyclic core of the Yohimbine and *Corynanthe* alkaloids showed potent inhibition *Tb*MetRS aminoacylation activity and *T. brucei* parasite proliferation. Testing of multiple hydroxyalkyl δ-lactam, δ-lactam, and piperidine analogs revealed one particular hydroxyalkyl δ-lactam derivative to be more effective against *T. brucei.* Still, they did not affect the aminoacylation activity of *Tb*MetRS (Table 1) (64).

Two bacterial MetRS inhibitors, REP3123 and REP8839, affected *P. falciparum* pathogen survival at various stages, viz., ring, trophozoite, and schizont (Table 1) (65) (Fig. 1). Three compounds, C1, C2, and C3, identified *in silico* were experimentally validated as they diminished protein translation by acting against *Pf*MetRS as they stopped the progression of parasite growth from the ring to the trophozoite stage (65) (Fig. 1). Class of imidazopyridine-containing compounds has shown promise against *C. parvum* and *C. hominis* infections in culture, likely *via* inhibition of *Cryptosporidium* MetRS (Table 1) (66). Compounds 2093, 2114, and 2259 showed the best *in vivo* activity; 2093 was not genotoxic. These compounds gradually stalled *C. parvum* infection in mouse models with no considerable side effects (66). This study points out that selectivity can be achieved over the human MetRS if treatment by these inhibitors is for short durations (*e.g.*, < 1 week). A new class inhibitor, compound-1717, a fluro-imidazopyridine, targets *Giardia intestinalis* MetRS and has ‘cidal’ anti-Giardia activity as it inhibited trophozoites growth (Fig. 1) at 500 nM with a therapeutic index of ∼100 (Table 1) (67). Compound-1717 satisfies Lipinski’s rule of 5 that determines the druggability of a molecule [Molecular mass less than 500 Da, high lipophilicity (expressed as partition coefficient LogP of less than 5), fewer than five hydrogen bond donors, less than ten hydrogen bond acceptors, and molar refractivity between 40–130]. It was later seen to be highly effective in clearing *Giardia* infection within 3 days at variable doses in a mouse model of giardiasis (68). Subsequently, another structurally novel class of inhibitors that contain a 4,6-diamino-substituted pyrazolopyrimidine core (the MetRS02 series) was identified (69). These compounds interestingly bind to an allosteric pocket in *L. major* MetRS. They also exhibit a noncompetitive mode of inhibition in enzymatic studies (Table 1) (69). Compound DDD806905 worked against promastigotes (Fig. 1) but did not work *in vivo*. MetRS is the most widely studied aaRS in this review, as it has been investigated in all pathogens discussed here except *T. gondii*.

### Phenylalanyl-tRNA synthetase (FRS)

Bicyclic azetidines have been explored as inhibitors for parasite phenylalanyl-tRNA synthetases (PheRS). *Cryptosporidium* PheRS was validated as the molecular target of bicyclic azetidines. The most potent compound, BRD7929 eliminated parasites *in vitro* exponentially, with a half-life of ∼9.5 h and ∼95 h was needed to kill 99.9% *C. parvum* parasites (Table 1) (70). Bicyclic azetidines have shown good selectivity as they eliminate parasites effectively in a mouse model with a once-daily dosing regimen. BRD7929 once-daily cured cryptosporidiosis in highly immunosuppressed mice and is thus promising for use in malnourished children and immunocompromised patients (70). Further, two compounds, bicyclic azetidines BRD7929 and BRD8494, were most potent across multiple stages of *C. parvum* growth *in vitro* across multiple stages, likely *via* inhibition of PheRS (Table 1) (71). BRD7929 was the most potent, possibly due to greater hydrophobicity. Series of bicyclic azetidines inhibit *T. gondii* growth *in vitro* and provide protection in a mouse model against acute and chronic toxoplasmosis (Table 1) (72). They are potent against tachyzoites (Fig. 1) at low nanomolar levels, and treatment of bradyzoites *in vitro* at EC_90_ concentrations leads to the complete killing of parasites. These compounds also exhibit better selectivity towards inhibiting *T. gondii* PheRS, thereby inhibiting parasite growth *in vitro* and *in vivo*. In particular, BRD7929 has an overall good bioavailability, potency, and desirable selective profile which has remained a major challenge for aaRSs inhibitors so far.

*P. falciparum* genome encodes for three different PheRS, wherein one complex *Pf*PheRS is localized to the cytosol and the apicoplast and a third unique *Pf*PheRS is localized in the mitochondria (73). A recent study revealed that BRD7929 had higher affinity and potent selective inhibition against *P. falciparum* cytoplasmic PheRS than the human PheRS (Table 1) (74). BRD7929 inhibits *P. falciparum* growth at nanomolar concentrations (EC_50_ 5 nM [Dd2 strain], 9 nM [3D7 strain]). It exhibited single-dose efficacy and promising pharmacokinetic properties in a mouse model. Three-dimensional structure of cytoplasmic *Pf*PheRS with BRD7929 reveals binding of the inhibitor at the L-Phe pocket and an adjacent auxiliary pocket which is interesting as it is a departure from most aaRSs where inhibitors occupy one or the other or both two substrate-binding sites (Table 1) (74). These drugs are shown to kill parasites *in vitro* and *in vivo* in all stages of the parasite life cycle (Fig. 1). Bicyclic azetidines are also competitive inhibitors of L-Phe in *P. vivax* PheRS, as BRD1389 binds similarly to the L-Phe pocket and an adjacent auxiliary pocket (75). Thus, in both these studies, *Pf* and *Pv* cytoplasmic PheRS show a similar binding mode.

PheRS has been explored as a druggable target only recently, and different bicyclic azetidines are now established as potent and promising inhibitors of PheRS from *Plasmodium*, *Cryptosporidium*, and *Toxoplasma*. PheRS holds a promise as an advanced target in parasites as it has shown the much-desired selectivity. Achieving selectivity remains a challenge in aaRSs since most of them share high homology with the human homologs and this is a major hindrance to successful progression from inhibitors to drugs. These inhibitors could be cross-tested for the other four pathogens discussed in this review.

### Prolyl-tRNA synthetase

Halofuginone, a synthetic derivative of febrifugine, binds to the L-Pro and tRNA sites in Plasmodium prolyl-tRNA synthetase (ProRS), confirming the enzyme to be a functional target of both febrifugine and halofuginone (76, 77, 78, 79, 80, 81). Febrifugine is well-established as a traditional Chinese herbal remedy for malaria fever for over a century (77). Halofuginone also kills *T. gondii* parasites suggesting the broad efficacy of this compound (78, 79). Halofuginone though highly potent in killing *Plasmodium* parasites causes cytotoxicity to host cells (77). Thus, many halofuginone and febrifugine derivatives with better safety profiles and improved therapeutic indices were designed for *Pf*ProRS (Table 1) (76, 77, 78, 79, 80, 81). Halofuginol, a new derivative of halofuginone was designed by modifying the linker region by replacing the ketone group with a secondary alcohol, is effective against the liver and blood stages (Fig. 1) of the parasite in a mouse model. Halofuginol demonstrated efficacy and tolerance in a *P. berghei*–infected mouse model (administrated orally or intraperitoneally at a 25 mg/kg dosage) (Table 1) (76).

In a recent study, “double drugging” of *T. gondii* ProRS by halofuginone and a novel ATP mimetic shows simultaneous binding at all three pockets in the active site since ATP mimetic L95 binds in the ATP site (82). Both L95 and halofuginone are effective at nM concentrations when used individually (82). Double drugging while managing dosage is a critical step towards likely achieving selectivity for ProRS and other aaRSs. Another study identified novel 1-(pyridin-4-yl)pyrrolidin-2-one derivatives as the cytoplasmic *Pf*ProRS inhibitors (83). Compound 1 and its enantiomer 1-S, when tested against resistant *Pf* strains and the development of liver schizonts (Fig. 1), showed potent low nanomolar activity. The slow killing and growth inhibition were seen in *Pf* and *Pv* field isolates. Thus, these derivatives show an encouraging off-target profile and oral efficacy in a *Pf* malaria murine model (83). Halofuginone and its derivatives are well-established and promising inhibitors of ProRS from *Plasmodium* and *Toxoplasma*. ProRS can be similarly explored in the remaining five of the seven pathogens discussed here.

### Threonyl-tRNA synthetase

Borrelidin is a likely inhibitor for *P. falciparum* threonyl-tRNA synthetase (ThrRS), belonging to class II, as it successfully inhibits the proliferation of parasites in culture and the asexual erythrocytic parasitic life-cycle, indicating cytosolic inhibition (Fig. 1) (Table 1) (84, 85, 86). However, no effect is seen in the apicoplast despite *Pf*ThrRS exhibiting dual localization (84, 85). Increased concentrations of L-Thr in culture reduced parasite sensitivity indicating Thr utilization and *Pf*ThrRS as the target for borrelidin. Subsequent studies showed *in vivo* effects of borrelidin as low doses cured mice of lethal rodent malaria infections caused by *Plasmodium yoelii* and possibly induced protective immune responses (85, 86). Borrelidin though an excellent inhibitor of *Pf*ThrRS (it shows antimalarial activity against drug-resistant Pf parasites with IC_50_ of 0.93 ng/ml) faces the challenge of cytotoxicity. In this direction, borrelidin analogs and borrelidin-like series are promising and show reduced host cytotoxicity (87). Khan *et al*. discovered novel inhibitors of *Pf*ThrRS by *in silico* screening using structural models revealing compounds with moderate inhibition of *P. falciparum* growth (18).

Eight potential *L. major* ThrRS inhibitors were screened using a prevalidated MNP library (20, 21). Two compounds inhibited the aminoacylation activity at ∼50%, and four (1438C, 1758C, 2059D, and 2096B) inhibited the activity by greater than 75%, which continued to perturb aminoacylation throughout experiments. Of these four, 2059D and 2096B also inhibit *L. major* AlaRS (Table 1). Borrelidin, well-established as a natural product inhibitor of bacterial ThrRS, also inhibits *T. brucei* ThrRS by inhibiting parasite growth (88). Further, knockdown of *T. brucei* ThrRS studies results in rapid cell death. Borrelidin shows a strong affinity for the *L. donovani* ThrRS (K_d_: 0.04 μM), and it also inhibits the promastigote stage of parasites (Fig. 1) (IC_50_: 21 μM) (Table 1) (89). Borrelidin and its analogs are well-established as inhibitors of ThrRS from *Plasmodium*, *Trypanosoma*, and *Leishmania* and they can be explored using established approaches in four of the seven pathogens discussed here.

### Tryptophanyl-tRNA synthetase

Two genes encode two tryptophanyl-tRNA synthetases (TrpRS), belonging to class I, in *T. brucei,* wherein the first recognizes the tRNA in the cytosol, and the second recognizes the tRNA inside the mitochondria (90). The second enzyme is needed for aminoacylation of the imported thiolated and the edited tRNA^Trp^ as it has a high substrate specificity (90). Parasite-specific subdomains with structural differences are seen in TrpRS from *Giardia* *lamblia, C. parvum, T. brucei*, and *B. histolytica,* and *P. falciparum* which can guide selective drug design (91, 92, 93, 94). The activation reaction mechanism is different in eukaryote *G. lamblia* compared to human TrpRS as three critical residues which stabilize interactions with a beta-hairpin are absent while retaining the overall dimer structure (91). An inhibitor of the bacterial TrpRS called indolmycin, a natural tryptophan analog, was explored against *P. falciparum* TrpRS. Indolmycin, isolated from the bacteria *Streptomyces griseus*, affects parasite growth by specifically inhibiting the apicoplast *Pf*TrpRS but not the cytosolic *Pf*TrpRS (Table 1) (95). The structure of the catalytic domain of cytoplasmic *Pf*TrpRS is available in complex with L-tryptophan (93).

### Tyrosyl-tRNA synthetase

The first three-dimensional structure of the *L. major* cytoplasmic tyrosyl-tRNA synthetase (TyrRS), belonging to class I, showed a pseudo-dimer with unique asymmetric domains and only a single functional, active site (near N-terminus) along with an anticodon site (near the C-terminus) (96). *L. donovani* TyrRS is characterized and validated as an essential enzyme (97). Fisetin (3,3′,4′,7-tetrahydroxyflavone), a flavonoid, inhibits parasite growth by inhibiting *Ld*TyrRS aminoacylation activity, as seen earlier for trypanosomal TyrRS (96, 97). The structure of *L. donovani* TyrRS in complex with tyrosyl-adenylate consists of two “pseudo” monomers, wherein the N-terminal monomer is able to perform amino acid activation, while the C-terminal monomer lacks this canonical function (98). The structure of the *Ld*TyrRS with bound to tyrosyl-adenylate revealed an “extra pocket” near the adenine-binding region, which is absent in human TyrRS (98). *P. falciparum* TyrRS is localized in the cytosol and is present in the infected erythrocytes (99). The extracellular activity of the *Pf*YRS was detected *via* mimicking host cytokines to then induce pro-inflammatory responses in the host (99).

A recent study has identified adenosine 5′-sulfamate (a close mimic of AMP), and then ML901 from Takeda Pharmaceutical compound library, which showed potent activity against all strains of *P. falciparum* and also showed 800- to 5000-fold selectivity towards the parasite (100). *In vivo* study in mice engrafted with *P. falciparum*–infected RBCs showed that a single dose [50 mg/kg intraperitoneal injection] can reduce parasitemia to baseline with no cytotoxicity. ML901 targets the *Pf*TyrRS by a unique “reaction hijacking” mechanism. The *Pf*YRS binds to ATP and tyrosine; the tRNA^Tyr^ then binds to the tyrosine-releasing AMP. This would normally make a “charged” tRNA but when inhibitor ML901 is present, this drug binds to the tyrosine of Tyr-tRNA^Tyr^ instead and the uncharged tRNA^Tyr^ is released thereby inhibiting the *Pf*TyrRS and stalling protein synthesis (100). This unique, promising approach may achieve the desired selectivity for aaRSs.

## Other structurally and functionally characterized aaRSs

### Aspartyl-tRNA synthetase

The first apo structure of the aspartyl-tRNA synthetase (AspRS), belonging to class II, from *E. histolytica,* was determined early on (101). The *T. brucei* genome encodes two separate genes for AspRS, though the cell contains a single tRNA^Asp^ isoacceptor. *In vitro* data shows that mitochondrial *Tb*AspRS2 aminoacylates cytosolic and mitochondrial tRNA^Asp^, whereas the cytosolic *Tb*AspRS1 only recognizes cytosolic tRNA^Asp^. Thus, cytosolic and mitochondrial tRNA^Asp^ are derived from the same nuclear gene product but are physically distinct, offering dual potential as drug targets (102). Structural data reveals that the N-terminus of the *P. falciparum* AspRS contains a motif that may provide a strong RNA binding to plasmodial AspRS, and plasmodial insertion is required for AspRS dimerization and thereby for its aminoacylation activity and other functions (103). AspRS has been reported in *Entamoeba*, *Trypanosoma*, and *Plasmodium* but is yet to be explored in five of seven pathogens discussed. AspRS has been reported in *Entamoeba*, *Trypanosoma*, and *Plasmodium* with 3D structures available from *E. histolytica* which can be utilized to develop first-ever inhibitors of AspRS.

### Glutamyl-tRNA synthetase

The transcript of GltX, one of the glutamyl tRNA synthetases (GluRS), is expressed during the asexual blood stages of *Babesia bovis*, which confirms that the complete bipartite signal is in control of directing the reporter protein into the apicoplast, a compartment distinct from the nucleus and the mitochondrion (104). Further, the Gln-tRNA(Gln) biosynthesis in the *Plasmodium* apicoplast is achieved by a vital indirect aminoacylation pathway, where GluRS is first targeted in the apicoplast in the blood stages as it glutamylates tRNA^Glu^ and tRNA^Gln^ (105). GluRS remains to be explored as no structure or inhibitor has been reported from the seven pathogens discussed here.

### Cysteinyl-tRNA synthetase

The cysteinyl-tRNA synthetase (CysRS), belonging to class I, is encoded by a single gene in *P. falciparum* and is dual-targeted to the cytosol and the apicoplast (106). Similar to the other well-studied dual location ThrRS, inhibition of CysRs will have dual effects, that is, first killing the parasites *via* inhibition of cytosolic translation and concurrently disrupting the apicoplast protein translation. CysRS has been partially studied in *P. falciparum* and remains to be explored in six of the seven pathogens discussed here, as no inhibitors have been discovered.

## Concluding remarks

The aaRSs are universally conserved enzymes essential for protein synthesis. Remarkable progress in the past two decades has thrust parasite-encoded aaRSs into focus as promising drug targets for many pathogens. Biochemical and structural studies have elucidated inhibition mechanisms that target various sites on these enzymes. Indeed, a remarkable example of double drugging of this enzyme family has also been validated wherein two unrelated and individually potent drugs co-bind to the ProRS, occupying all the substrate-binding sites on the enzyme (82). There is a wealth of knowledge on inhibitor identification, design, and development against numerous eukaryotic parasite aaRSs. These studies are based on various technologies that include *in silico* docking, high-throughput screening, enzyme inhibition assays, chemical modifications of hit compounds, and animal models. The libraries of hit compounds generated so far for individual pathogens can be tested across many more eukaryotic pathogens allowing cross-usage of existing and new inhibitors. These parasite aaRSs tend to have conserved 3D structures and conserved catalytic sites in their aminoacylation domains. Evaluating sequence conservation in the inhibitor-binding residues in drug bound complexes may validate new hit compounds which could be used to target more than one pathogen. We have, in an earlier study, proposed “STOPP”, that is, structure-based targeting of orthologous pathogen proteins (107). Essential enzymes that are conserved (between hosts and pathogens and within different parasites) have the potential to be excellent drug targets if new compounds and chemical entities can differentiate subtle sequence/structure variations in the binding region. This knowledge can be leveraged when synthesizing novel chemical entities. Such structure-based targeting of orthologous proteins will jump-start inhibitor discovery across pathogen-encoded aaRSs.

Resistance to antiparasitic drugs remains a significant issue and warrants special focus during the preclinical stages of inhibitor development. Early evaluations of inhibitors can put them on a robust path and make them promising scaffolds. Due to the universal nature of aaRSs enzymes, selectivity remains a challenge for developing successful drugs against parasite aaRSs even though these parasites, in some cases, are evolutionarily distinct and cause different diseases. The selectivity of an inhibitor towards parasite aaRS and not the human host aaRS is crucial as this results in host cytotoxicity. This issue of selectivity warrants attention and requires a thorough understanding of the structural underpinnings for specific drug design. The data consolidated in this work will pave the way for further dissection of aaRSs from eukaryotic pathogens and will steer the modification of promising inhibitors and scaffolds into selective drug-like compounds. Parasite-encoded aaRSs are undoubtedly exciting and promising druggable targets that warrant continued scrutiny for the development of anti-infective drugs.

## Conflict of interest

All authors declare that they have no conflicts of interest with the contents of this article.
